# The Past, Present, and Future of Immune Repertoire Biology – The Rise of Next-Generation Repertoire Analysis

**DOI:** 10.3389/fimmu.2013.00413

**Published:** 2013-11-27

**Authors:** Adrien Six, Maria Encarnita Mariotti-Ferrandiz, Wahiba Chaara, Susana Magadan, Hang-Phuong Pham, Marie-Paule Lefranc, Thierry Mora, Véronique Thomas-Vaslin, Aleksandra M. Walczak, Pierre Boudinot

**Affiliations:** ^1^UPMC University Paris 06, UMR 7211, Immunology-Immunopathology-Immunotherapy (I3), Paris, France; ^2^CNRS, UMR 7211, Immunology-Immunopathology-Immunotherapy (I3), Paris, France; ^3^INSERM, UMR_S 959, Immunology-Immunopathology-Immunotherapy (I3), Paris, France; ^4^AP-HP, Hôpital Pitié-Salpêtrière, CIC-BTi Biotherapy, Paris, France; ^5^AP-HP, Hôpital Pitié-Salpêtrière, Département Hospitalo-Universitaire (DHU), Inflammation-Immunopathology-Biotherapy (i2B), Paris, France; ^6^Institut National de la Recherche Agronomique, Unité de Virologie et Immunologie Moléculaires, Jouy-en-Josas, France; ^7^IMGT^®^, The International ImMunoGeneTics Information System^®^, Institut de Génétique Humaine, UPR CNRS 1142, Université Montpellier 2, Montpellier, France; ^8^Laboratoire de Physique Statistique, UMR8550, CNRS and Ecole Normale Supérieure, Paris, France; ^9^Laboratoire de Physique Théorique, UMR8549, CNRS and Ecole Normale Supérieure, Paris, France

**Keywords:** diversity analysis, immune receptors, next-generation sequencing, modeling, statistics, gene nomenclature, B cell repertoire, T cell repertoire

## Abstract

T and B cell repertoires are collections of lymphocytes, each characterized by its antigen-specific receptor. We review here classical technologies and analysis strategies developed to assess immunoglobulin (IG) and T cell receptor (TR) repertoire diversity, and describe recent advances in the field. First, we describe the broad range of available methodological tools developed in the past decades, each of which answering different questions and showing complementarity for progressive identification of the level of repertoire alterations: global overview of the diversity by flow cytometry, IG repertoire descriptions at the protein level for the identification of IG reactivities, IG/TR CDR3 spectratyping strategies, and related molecular quantification or dynamics of T/B cell differentiation. Additionally, we introduce the recent technological advances in molecular biology tools allowing deeper analysis of IG/TR diversity by next-generation sequencing (NGS), offering systematic and comprehensive sequencing of IG/TR transcripts in a short amount of time. NGS provides several angles of analysis such as clonotype frequency, CDR3 diversity, CDR3 sequence analysis, V allele identification with a quantitative dimension, therefore requiring high-throughput analysis tools development. In this line, we discuss the recent efforts made for nomenclature standardization and ontology development. We then present the variety of available statistical analysis and modeling approaches developed with regards to the various levels of diversity analysis, and reveal the increasing sophistication of those modeling approaches. To conclude, we provide some examples of recent mathematical modeling strategies and perspectives that illustrate the active rise of a “next-generation” of repertoire analysis.

## Introduction

T and B cell repertoires are collections of lymphocytes, each characterized by its antigen-specific receptor. The resources available to generate the potential repertoires are described by the genomic T cell receptor (TR) and immunoglobulin (IG) loci. TR and IG are produced by random somatic rearrangements of V, D, and J genes during lymphocyte differentiation. The product of the V-(D)-J joining, called the complementarity determining region 3 (CDR3) and corresponding to the signature of the rearrangement, binds the antigen and is responsible for the specificity of the recognition. During their differentiation, lymphocytes are subjected to selective processes, which lead to deletion of most auto-reactive cells, selection, export, and expansion, of mature T and B cells to the periphery. Primary IG and TR repertoires are therefore shaped to generate the available peripheral or mucosal repertoires. In addition, several different functional T and B cells subsets have been identified, with differential dynamics and antigen-specific patterns. These available repertoires are dramatically modified during antigen-driven responses especially in the inflammatory context of pathogen infections, autoimmune syndromes, and cancer to shape actual repertoires. When considering the importance of efficient adaptive immune responses to get rid of infections naturally or to avoid auto-reactive damages, but also for therapeutic purposes such as vaccination or cell therapy, one realizes the relevance of understanding how lymphocyte repertoires are selected during differentiation, from ontogeny to aging, and upon antigenic challenge. However, immune repertoires of expressed antigen receptors are built by an integrated system of genomic recombination and controlled expression, and follow complex time-space developmental patterns. Thus, an efficient repertoire analysis requires both (1) methods that sample and describe the diversity of receptors at different levels for an acceptable cost and from a little amount of material and (2) analysis strategies that reconstitute the best multidimensional picture of the immune diversity from the partial information provided by the repertoire description as reviewed in Ref. (1). In the following sections, we summarize technologies developed over the past decades to describe lymphocyte repertoires and we present the growing number of analysis tools, evolving from basic to sophisticated statistics and modeling strategies with regards to the level of complexity of the data produced.

## Methods Developed to Describe the IG and TR Repertoires

B and T lymphocyte repertoires can be studied from different lymphoid tissues and at various biological levels, such as cell membrane or secreted proteins, transcripts or genes, according to the techniques used. Fluorescence microscopy or flow cytometry techniques allow to track and sort particular cell phenotypes and to quantify the expressed repertoire at the single-cell level with V subgroup-specific monoclonal antibodies. Alternatively, the IG or TR diversity may be also analyzed using proteomics methods from either the serum (for IG) or dedicated cell extracts. Finally, molecular biology techniques assess the repertoire at the genomic DNA or transcriptional levels, qualitatively and/or quantitatively.

### Analysis of IG and TR repertoires at the protein level

#### Flow cytometry single-cell repertoire analysis

The frequency of lymphocytes expressing a given IG or TR can be determined using flow cytometry when specific monoclonal antibodies are available. This technique allows for the combined analysis of the antigen receptor and of other cell surface markers. Currently, using flow cytometry, up to 13 parameters can be routinely studied at once, reaching 20 parameters with the last generation flow cytometers and 70–100 parameters with mass cytometry (2). Seminal studies in mice using specific anti-TRBV antibodies have led to the characterization of the central tolerance selection processes that occur in thymus (3–5). Later on, a comprehensive description of the human TRBV repertoire was setup (6), when monoclonal antibodies became available for most of the TRBV subgroups. Repertoire analysis with flow cytometry provides a qualitative and quantitative analyses of the variable region, often done on heterogeneous cell populations, in order to decipher, for example, selection events related to aging, perturbations, and treatments (7). However, this technology is naturally limited by the availability of specific monoclonal antibodies, and does not address more detailed issues such as junction diversity. Furthermore, polymorphism of the IG or TR genes (8, 9) may constitute a serious limitation for a systematic survey using these approaches.

#### Proteomic repertoire analysis for serum immunoglobulins

Recent developments of proteomics tools now offer sensitivity levels applicable to IG repertoire analysis. Such a description at the protein level takes into account all post transcriptional and translational modifications.

##### PANAMA-blot technology

A semi-quantitative immunoblot, called the PANAMA-blot technique (10), allows for the identification of the antibody reactivities present in collection of sera (or cell culture supernatant) against a given source of antigens (10–12). Briefly, a selected source of antigens is subjected to preparative SDS-PAGE, transferred onto nitrocellulose membranes, then incubated with the serum to be tested allowing for the revelation of the bound antibodies using an appropriate secondary antibody coupled to alkaline phosphatase. Computer-assisted analysis of the densitometric profiles allows for the rescaling and the quantitative comparison of patterns of antibody reactivity from individuals in different groups. A large amount of data is generated when testing a range of sera against various sources of antigens. Statistical analyses are included in the PANAMA-Blot approach (as described further). This global analysis helped to reveal that the IgM repertoire in mice is selected by internal ligands and independent of external antigens (13).

This method can also lead to identify IG reactivity patterns specific for a type of pathology or clinical status and has been applied to both fundamental and clinical analysis. In particular, it was used to analyze human self-reactive antibody repertoires and their potential role for down-modulating autoimmune processes (14–16).

##### Antigen micro-array chips

More recently, antigen micro-array-based technology coupled to a complex two-way clustering bioinformatics analysis was developed to evaluate the serum repertoire antibodies from diabetes-prone individuals and revealed their predictive or diagnostic value. In brief, a range of antigens (proteins, peptides, nucleotides, phospholipids…) were plated onto glass plates and incubated with sera from individuals (human diabetes patients or mice in an experimental model of diabetes). The intensity of reactivity of the serum IG for each peptide was determined and scored against the control reactivity. Clustering analysis was then implemented to determine a potential antigen signature that significantly sorts out diabetes from non-diabetes individuals. In this way, it was found that the patterns of IgG antibodies expressed early in male NOD mice can mark susceptibility or resistance to diabetes induced later and that it is different than the pattern characteristic of healthy or diabetic mice after disease induction (17). Similarly, this clustering approach was applied in humans to successfully separate human subjects that are already diabetic from healthy people (18).

### Repertoire analysis at the genomic DNA level

Other strategies that cover IG or TR repertoire analyses have been developed at the genomic DNA level. Firstly, CDR3 spectratyping studies (detailed in the following section) have been carried out at the DNA level mostly to address issues related to B or T cell development (19, 20). More recently, an original multiplex genomic PCR assay coupled to real-time PCR analysis was developed to provide a comprehensive description of the mouse T cell receptor alpha (TRA) repertoire during development (21). Although these approaches can be applied to all IG isotypes and TR, they have not been used as much as transcript CDR3 spectratyping due to sensitivity and heterozygosity issues.

Immunoglobulin or T cell receptor repertoires can also be assessed by following the diversity of rearrangement deletion circles. Since they are produced by the V-(D)-J recombination machinery when the joint signal is formed and diluted in daughter cells, they give a good representation of recently generated T or B cells. This technique has been particularly useful for describing the restoration of T cell diversity following highly active antiretroviral therapy in HIV-infected patients (22) and has been used to model thymic export (23, 24) as well as to demonstrate continued contribution of the thymus to repertoire diversity, even in older individuals (25). It also reveals that thymic output is genetically determined, and related to the extent of proliferation of T cells at DN4 stage in mice (26). However, their analysis does not provide much insight into the level of diversity since the signal joint does not vary for a given combination of genes. Therefore, the interest of such analyses is reached when combined with CDR3 spectratyping analyses to know whether a repertoire perturbation is rather attributable to newly produced T cells or peripheral T cell proliferation.

### V-(D)-J junction analysis of IG and TR transcript repertoires

Original molecular-based strategies for analyzing repertoire diversity relied on cloning and hybridization of molecular probes specific for IGHV gene subgroups first by RNA colony blot assay (27). This led to the observation that IGHV gene usage is characteristic of mouse strain and is a process of random genetic combination by equiprobable expression of IGHV genes (28). The study of selection processes revealed that the IGHV region-dependent selection determines clonal persistence of B cells (29) and that selection with age leads to biased IGHV gene expression (30).

*In situ* hybridization on single-cells revealed that during mouse ontogeny and early development of B cells in bone marrow, there is a non-random position-dependent IGHV gene expression, favoring D-proximal IGHV gene subgroup usage (31). Thereafter, sequencing of PCR-amplified cDNA collections were obtained from samples of interest. Although fastidious, these early studies have been useful in defining the basis of human IG and TR repertoires in terms of overall distribution, CDR3-length distribution, and V-(D)-J use (32–35), sometimes leading to the identification of new IG or TR genes. Later, more practical techniques have been developed for large-scale analysis of lymphocyte repertoires, such as quantitative PCR, micro-array, and junction length spectratyping, as described below.

#### Quantitative RT-PCR for repertoire analysis

In parallel to qualitative CDR3 spectratyping techniques (see section below), quantitative PCR strategies were developed (36). Coupling the two techniques for all V domain-C region combinations provides a complete qualitative and quantitative picture of the repertoire (37–39) described by up to 2,000 measurements per IG isotype or TR for one sample. With the development of real-time quantitative PCR, this approach opened the possibility for a more precise evaluation of repertoire diversity (39–41). Complementary tools have been also developed in order to allow normalization of spectratype analysis such as studies by Liu et al. (42) and Mugnaini et al. (43).

Matsutani et al. (44) developed another method to quantify the expression of the human TRAV and TRBV repertoires based on hybridization with gene specific primers coated plates. The cDNA from PBMC extracted RNA are ligated to a universal adaptor which allows for a global amplification of all TRAV or TRBV cDNAs. The PCR products are then transferred onto microplates coated with oligonucleotides specific for each TRAV or TRBV regions, and the amount of hybridized material is quantified. This technique was used to analyze the TR repertoire diversity of transplanted patients (45) and adapted to the study of mouse TRAV and TRBV repertoires (46). VanderBorght et al. also developed a semi-quantitative PCR-ELISA-based method for the human TRAV and TRBV repertoire analysis (38). The combined usage of digoxigenin (DIG)-coupled nucleotides and DIG-coupled reverse TRAC or TRBC primers allowed for a quantitative measurement of the amount of amplified DNA by a sandwich ELISA.

Du et al. (47) later setup a megaplex PCR strategy to characterize the antigen-specific TRBV repertoire from sorted IFNγ-producing cells after *Mycobacterium* infection. The clonotypic TRBV PCR products were used for Taqman probes design to quantify the expression of the corresponding clonotypes from ATLAS-amplified SMART cDNAs.

#### Direct measurement of lymphocyte diversity using micro-arrays

Another technology, similar to the one just discussed, has been developed by the group of Cascalho et al. which allows for a direct measurement of the entire population of lymphocyte-receptors. This is accomplished by hybridization of lymphocyte-receptor specific cRNA of a lymphocyte population of interest to random oligonucleotides on a gene chip; the number of sites undergoing hybridization corresponds to the level of diversity. This method was validated and calibrated using control samples of random oligonucleotides of known diversity (1, 10^3^, 10^6^, 10^9^) (48, 49) and successfully demonstrated that central and peripheral diversification of T lymphocytes is dependent on the diversity of the circulating IG repertoire (49, 50). Similarly, a highly sensitive micro-array-based method has been proposed to monitor TR repertoire at the single-cell level (51).

#### CDR3 spectratyping techniques

##### Immunoscope technology

Among various techniques used to analyze the T or B cell repertoires, Immunoscope, also known as CDR3 spectratyping (52, 53) consists in the analysis of the CDR3-length usage so that antigen-specific receptor repertoires can be described by thousands of measurements. In the case of naive murine repertoires, T cell populations are polyclonal and analysis typically yields eight-peak regular bell-shaped CDR3 displays (wrongly assumed to be Gaussian), each peak corresponding to a given CDR3-length. When an immune response occurs, this regular polyclonal display can be perturbed: one can see one or several prominent peaks that correspond to the oligoclonal or clonal expansion of lymphocytes. A complete description of this technique and its applications to clinical studies has been published elsewhere (54).

In the original Immunoscope publication, Cochet et al. (55) analyzed the T cell repertoire after the immunization of mice with the pigeon cytochrome c. They provided the first description of an *ex vivo* follow-up of a primary T cell specific response in a mouse model. Their second paper analyzed the average CDR3-lengths as a function of TRBV-TRBJ combinations. In particular, the authors found a correlation between TRBV CDR1 and major histocompatibility (MH) haplotype (52). This group later published a large amount of original studies in various models such as lymphocyte development (40, 56–63), kinetics of antigen-specific responses (64–67), viral infection (68, 69), autoimmunity (70, 71), tumor-associated disease (72), and analysis of allogeneic T cell response and tolerance after transplantation (73). Notably, the combination of CDR3 spectratyping with flow cytometry-based IG or TR V frequency analysis provides a more comprehensive assessment, such as in Pilch et al. (74). For example, such an approach revealed the constriction of repertoire diversity through age-related clonal CD8 expansion (75). Similarly, a combination of CDR3 spectratyping, flow cytometry, and TR deletion circle analysis has allowed to define age-dependent incidence on thymic renewal in patients (76) or to evaluate the effects of caloric restriction in monkeys to preserve repertoire diversity (77). CDR3-length spectratyping was also used in other models, such as rainbow trout, to analyze TRB repertoire and its modifications induced by viral infection (78–80). While no tool such as monoclonal antibodies to T cell marker(s) was available in this model, this approach demonstrated that fish could mount specific T cell responses against virus, which could be found in all individuals (public clonotypes) or not (private clonotypes). Similar strategies, developed by other groups (81) and following the same approach in parallel, analyzed the IG repertoire in *Xenopus* at different stages of development, describing a more restricted IG junction diversity in the tadpole compared to the adult.

Gorski et al. (82) developed their own CDR3 spectratyping technique to analyze the complexity and stability of circulating αβT cell repertoires in patients following bone marrow transplantation as compared to normal adults. They showed that repertoire complexity of bone marrow recipients correlates with their state of immune function; in particular, individuals suffering from recurrent infections associated with T cell impairment exhibited contractions and gaps in repertoire diversity. The detailed procedure for this technique has been published in Maslanka et al. (83). A variation of this technique has been reported later by Lue et al. (84), relying on a compact glass cassette, a simpler device than the usual automated plate DNA sequencers.

##### Alternative technologies

Alternative CDR3 spectratyping techniques have been described such as single-strand conformation polymorphism (85–87) and heteroduplex analysis (88–91). These methods differ from the CDR3 spectratyping/Immunoscope technique mostly in the way PCR products are analyzed by performing non-denaturing polyacrylamide electrophoresis. The main advantage of these techniques is a more direct assessment of clonal expansion since PCR products migrate according to their conformation properties; therefore, presence of a predominant peak is strongly indicative of clonality when a smear migration pattern indicates polyclonality. However, these techniques have been less widely used probably because of the difficulty to make clear correlations between the expanded peaks across samples.

Another original alternative technique has been described by Bouffard et al. (92), analyzing products obtained after *in vitro* translation of PCR-amplified TR-specific products by isoelectric focusing. With this technique, clonality can also directly be assessed by looking at the obtained migration profile.

### IG/TR rearrangement sequencing: From cloning-based- to next-generation-sequencing

In order to get a better description of IG/TR diversity at the nucleotide sequence level, thus providing fine-tuned description of the actual diversity, Sanger sequencing approaches relying on bacterial cloning of rearrangements were performed in physiological conditions globally (60, 93–99) or partially to characterize particular expansions identified by other technologies such as CDR3 spectratyping (40, 59, 100–102), flow cytometry (103). They were also used in pathological/infectious conditions (104–107) sometimes leading to antigen-specific T cell TR identification and quantification through the combination of antigen-specific T cell stimulation and cytometry-based cell sorting, anchor-PCR, and bacterial cloning-based sequencing (108).

These studies pioneered the description of the repertoire and provided fruitful information regarding the extent and modification of the diversity. However, besides being time and cost-extensive, such approaches have allowed for the analysis of 10^2^–10^3^ sequences, far under the estimated diversity reaching 10^6^–10^7^ unique clonotypes in mice and humans (40, 59, 109).

In the last decade, DNA sequencing technologies have made tremendous progresses (110) with the development of so called next-generation sequencers, already reaching four generations (111). Those instruments are designed to sequence mixtures of up to millions of DNA molecules simultaneously, instead of individual clones separately. Second generation sequencers became affordable in the last 5 years and have been used for immune repertoire analysis, starting with the seminal work of Weinstein et al. (112) where the IG repertoire of Zebrafish has been described by large-scale sequencing. Consequently, exploratory works by other groups provided an overview of the complex sequence landscape of immune repertoires in humans (113–118). More recent work aimed at addressing fundamental questions such as lineage cells commitment (119–122), generation of the diversity processes (123–125), and diversity sharing between individuals (126, 127). Finally, the power of this technology has been validated in the clinic as well (128, 129).

As seen above for other technologies, combinations of approaches have been applied to NGS. Notably, deep sequencing has been used in combination with CDR3-length spectratyping by some groups to study human (130) or rainbow trout IG (131) repertoire modifications after vaccination against bacteria or viruses. In the latter, pyrosequencing performed for relevant VH/Cμ or VH/Cτ junctions identified the clonal structure of responses, and showed, for example, that public responses are made of different clones identified by (1) distinct V-(D)-J junctions encoding the same protein sequence or (2) distinct V-(D)-J sequences differing by one or two conservative amino acid changes (131) as described for public response in mammals (132, 133). These studies showed that NGS and traditional spectratyping techniques lead to remarkably similar CDR3 distributions.

Several NGS have been developed in the past years using different sequencing technologies characterized with different speed, deepness and read length. Metzker thoroughly reviewed their principles and properties (134). Among them, three platforms, all offering benchtop sequencers with reduced cost and setup, fit with immune repertoire analysis in terms of read length and deepness. The 454/Roche platform uses pyrosequencing technology (135), which combines single nucleotide addition (SNA) with chemoluminescent detection on templates that are clonally amplified by emulsion PCR and loaded on a picotiter plate. Pyrosequencing currently has a 500 bp (GS Junior) to 700 bp (GS FLX) sequencing capacity with a respective deepness of 150,000–3,000,000 reads per run (134). The Illumina/Solexa platform technology is based on cyclic reversible termination (CRT) sequencing (an adaptation of Sanger sequencing) performed on templates clonally amplified on solid-phase bridge PCR. Protected fluorescent nucleotides are added, imaged, delabeled, and deprotected cyclically (134), providing a deeper sequencing (from 15 to 6 billion reads per run for the MiSeq to the HiSeq2500/2000) of shorter reads (100–250 bp for the very recent MiSeq) with the possibility to perform pair-end sequencing (two-side sequencing) to increase the read length after aligning the generated complementary sequences. A more recent platform, Ion Torrent/Life Technologies using an imaging free detection system may open a new era in terms of deepness (one billion reads per run) of 200 bp reads (136) in a very short time and on a benchtop sequencer. Importantly, depending on the technology, errors due to the PCR-based sample preparation and the sequencing are of major concern. Bolotin et al. (137) evaluated this issue on TR repertoire analysis of the same donor performed on the three platforms described previously; algorithms for error correction have been developed. Indeed, PCR- and sequencing-related errors represent the major concern for immune repertoire diversity analysis as they may generate artificial diversity. Illumina and 454 appear to be the most robust technologies, with Illumina having the highest throughput and 454 generating the longest reads. The currently available Ion Torrent platform, although very promising, has been shown to display the highest rate of errors in TR (137) and bacterial DNA (138) sequencing. However, such error corrections must be used with caution since they may inadvertently underestimate repertoire diversity by removing rare sequences.

With the power of such approach for genomics and transcriptomics studies in general, constant improvements are achieved to increase the sequencing deepness and read length as well as to reduce the cost, therefore offering multitude of biological explorations (139). NGS now permits a comprehensive and quantitative view of IG and TR diversity by combining and improving the sensitivity of classical approaches with accurate and large-scale sequencing. NGS has the power to identify IG or TR specific for given antigens (in combination with antigen-specific assays) and to define more complex signatures (i.e., TR sets) related to disease and/or treatment from heterogeneous T and B cell populations. Still, most of the deep sequencing efforts have been limited to only one chain of the receptor at the repertoire level (usually the β chain for TR and the heavy chain for IG). Indeed, current high-throughput approaches do not allow one to assign which combination of chains (TRA and TRB, or IGH and IGK or IGL) belong to which cell (140). A recent development by DeKosky et al. proposed a reasonably high-throughput technology to assess massively paired IG VH and VL from bulk population (141). In parallel, Turchaninova et al. (142) have proposed a similar approach for the paired analysis of the TRA and TRB chains. The parallel development of high-throughput microfluidic-based single-cell sorting will certainly push forward new developments in the field (143).

However, despite the technological advance, studies so far have mainly reported CDR3 counting and identification of major expansions. The complexity of immune repertoires is still a matter that such approach cannot completely overcome, due to the paucity of powerful analytical methods. Besides data management tools, studies are now starting to extract most of the benefit from such approach to model the immune repertoire diversity and dynamics (144), an approach that may help in understanding the interplay between cells and repertoire shaping. Accurate and powerful statistical analyses are required to manage such amount of information. Current state will be reviewed in the following sections.

## Potential and Genomic Repertoires: A Question of Ontology and Orthology

Immune repertoires *sensu stricto* are expressed by lymphocyte clones, each carrying a single receptor for the antigen. Such receptors comprise IG and TR in jawed vertebrates (8, 9) and VLR in Agnathans (145). The sequences of these receptors are available in databases such as GenBank or EMBL, which are difficult to use for transversal studies due to inconsistent annotation. The IMGT^®^ information system (see below) has largely solved this problem setting standardized gene nomenclatures, ontologies and a universal numbering of the IG/TR V and C domains, thus giving a common access to standardized data from genome, proteome, genetics, two-dimensional, and three-dimensional structures (146). The accuracy and the consistency of the IMGT^®^ data are based on IMGT-ONTOLOGY, the first, and so far, unique ontology for immunogenetics and immunoinformatics (147).

With the development of high-throughput sequencing, large numbers of new sequences of antigen receptor genes have become available, which can be classified into different categories: genomic sequences of IG or TR (in germline configuration in genome assemblies) or fragments of IG/TR transcripts, containing the CDR3 or not. Also, these datasets can be produced from species newly sequenced, as well as from new haplotypes of well-described species.

The annotation of such sequences remains an open question. Manual annotation is not applicable, and no good automated approach has been validated yet. A relevant annotation of these massive datasets will require the integration of genomic and expression data with existing standardized description charts, as offered by IMGT^®^. A standardized annotation is an important issue since it facilitates the re-utilization of datasets and comparison of analyses. Thus, the description of IG and TR polymorphisms, the integration of repertoire studies with structural features of antigen-specific domains, and even the usage of new genes in genetic engineering rely on a common standard for nomenclature, numbering, and annotation (147).

To take advantage of the current standards that have been established from classical sequencing data during the last 25 years, new, fast, reliable, and human-supervised annotation methods will have to be developed, integrating directly high-throughput sequence information from the increasing number of deep sequencing platforms and technologies, at different genetic levels (genome, transcriptome, clonotype repertoires). Along this line, IMGT/HighV-QUEST offers online tools to the scientific community for the analysis of long IG and TR sequences from NGS (148).

Special attention can be paid to the orthology/paralogy relationships between similar antigen receptor genes from different species. These characteristics are essential to understand the dynamics of IG and TR loci. In fact, with many important lymphocyte subsets characterized by canonical/invariant antigen receptors, such relationships are critical to transfer functional knowledge between models. Importantly, the phylogenetic analyses required to reconstitute the evolution of antigen receptor genes are based on multiple alignments, the quality of which is highly dependent on common numbering and precise annotation of sequences.

As far as immune repertoires are characterized by the diversity of receptors specifically binding antigen/pathogen motifs to initiate a defense response, they might not be limited to lymphocyte diversifying receptors, e.g., IG, TR, and VLR. The particularity of these systems is a somatic diversification combined to a clonal structure of the repertoire, each lymphocyte clone expressing the product of a recombination/hypermutation and/or conversion process. However, many other arrays of diverse receptors binding or sensing pathogens have been discovered in metazoans, in invertebrates as well as in vertebrates.

In some cases, their diversity is really “innate,” i.e., encoded in the genome as multiple genes produced by duplications. Fish NLR, finTRIMs, and NITR, primate KIR, chicken CHIR, or TLR in sea urchin, constitute good examples of such situations. While these repertoires may appear as relatively limited, polymorphism within populations, and differential expression of receptors per cell upon stimulation represent complex issues, which fall well into “traditional” repertoire approaches.

In other cases, receptors are subject to diversification processes much faster than gene duplication, which does not comply with a clonal selection pattern. The best examples are probably the DSCAM in arthropods, which hugely diversify by alternative splicing of exons encoding half-IgSF domains (149, 150), and the FREP lectins in mollusks, of which sequences are highly variable at the population level, and even between parents and offspring produced by auto-fecundation (151).

The number of such “innate” repertoires which are not expressed by clonally selected lymphocytes will likely increase with deep sequencing of new genomes/transcriptomes, as illustrated by a recent report from mussel (152). A good example of the importance of a proper structural description of key domains of receptors is provided by the extensive analysis of LRR motifs in studies on TLR evolution (153, 154). Further insights into the functions of such diverse proteins will be provided by the characterization of their expressed (available) repertoire, at different levels such as single-cells, cell populations, and animal populations.

Such analyses will require precise identification of genes and sequences as well as mutations, and a standardized approach of nomenclature and structural description will be as useful as it is for the vertebrate IG and TR sequences. Importantly, these receptors are made of a small number of structural units, such as IgSF domain or LRR domains, which suggests that standardized system(s) for sequence annotation could be developed following IMGT standards (155).

## Statistical Analysis and Modeling of Immune Repertoire Data

### Statistical repertoire analysis

The description of the repertoire modifications using flow cytometry or Immunoscope provided clear-cut and detailed insight into the clonal expansion processes during the responses against a defined antigen (64, 66). However, it is difficult to identify the relevant alterations of the repertoires in more complex situations such as pathogen infections or variable genetic backgrounds. For example, it appeared impossible to identify all significant modifications of TRB Immunoscope profiles during cerebral malaria by direct ocular comparison (107). Different methods were therefore developed to extract from IG and TR repertoire descriptions the relevant information, to encode it as numerical tables and to analyze them with statistical models.

#### CDR3 spectratype perturbation indices

Since the initial description of the CDR3 spectratyping technique, different scoring indices were developed or derived from the literature: “relative index of stimulation” (RIS) (55), “overall complexity score” (156), Reperturb (157), “complexity scoring system” (158), COPOM (159), Oligoscore (160), TcLandscape (161), “spectratype diversity scoring system” (162), Morisita-Horn index and Jaccard index (95–97), “absolute perturbance value” (163). A comparative review of such scoring strategies was published by Miqueu et al. (164).

In particular, the perturbation index Reperturb was developed by Gorochov et al. to perform TR repertoire analysis in HIV patient during progression to AIDS and under antiretroviral therapy. They could show drastic restrictions in the CD8^+^ T cell repertoire at all stages of natural progression that persisted during the first 6 months of treatment. In contrast, CD4^+^ T cell repertoire perturbations correlated with progression to AIDS with a return to a diversified repertoire in good responders to treatment (157).

Soulillou et al. refined this approach by combining the qualitative information obtained with usual CDR3 spectratyping with quantitative information of TRBV usage obtained by real-time quantitative PCR. They devised a four-dimension representation that represents TRBV subgroups, CDR3-length and percentage of TRBV use on three axis chart in addition to a color-coded representation of the CDR3 profile perturbation. Using this original approach, they were able to show that graft rejection is associated with a vigorous polyclonal accumulation of TRBV mRNA among graft-infiltrating T lymphocytes, whereas in tolerated grafts T cell repertoire is strongly altered (161, 165). Their study puts the emphasis on the importance of not only qualitative but also quantitative analysis of lymphocyte repertoires.

#### Platforms for repertoire data management and statistical analysis

Several platforms have been developed and rely mostly on CDR3 spectratyping and sequencing data, with recent developments to manage and analyze NGS data.

The ISEApeaks strategy and software were developed in order to satisfy the needs for efficient automated electrophoresis data retrieval and management (160, 166). ISEApeaks extracts peak area and length data generated by software used to determine fragment intensity and size. CDR3 spectratype raw data, consisting of peak areas and nucleotide lengths for each V-(D)-J-C combination, is extracted, smoothed, managed, and analyzed. The repertoires of different samples are gathered in a peak database and CDR3 spectratypes can be analyzed by different perturbation indices and multivariate statistical methods implemented in ISEApeaks. We have applied our ISEApeaks strategy in several studies. In an experimental model of cerebral malaria, we established a correlation between the quality of TR repertoire alterations and the clinical status of infected mice, whether they developed cerebral malaria or not (107). We contributed to the characterization of the membrane-associated *Leishmania* antigens (MLA) that stimulates a large fraction of naive CD4 lymphocytes. Repertoire analyses showed that MLA-induced T cell expansions used TR with various TRBV rearrangements and CDR3 lengths, a feature closer to that of polyclonal activators than of a classic antigen (167). We also revealed repertoire age-related perturbations in mice (7). ISEApeaks functions for statistical analysis was successfully applied to analyze the TR repertoire in fish as shown by our detailed analysis of the TRB repertoire of rainbow trout IELs, performed in both naive and virus-infected animals. Rainbow trout IEL TRBV transcripts were highly diverse and polyclonal in adult naive individuals, in sharp contrast with the restricted diversity of IEL oligoclonal repertoires described in birds and mammals (102). More recently, our study of the CD8^+^ and CD8^−^ αβ T cell repertoire suggests different regulatory patterns of those T cell patterns in fish and in mammals (168). ISEApeaks was also used to implement a new statistically based strategy for quantification of repertoire diversity (159).

Kepler et al. described another original statistical approach for CDR3 spectratype analysis, using complex procedures for testing hypotheses regarding differences in antigen receptor distribution and variable repertoire diversity in different treatment groups. This approach is based on the derivation of probability distributions directly from spectratype data instead of using *ad hoc* measures of spectratype differences (169). A software (called SpA) implementing this method has been developed and made available online (170). This approach has been used in a longitudinal analysis of TRBV repertoire during acute GvHD after stem cell transplantation (171).

Another group (163) reported the development of a new software platform, REPERTOIRE, which allows handling of CDR3 spectratyping data. This software implements a perturbation index based upon an expected normal Gaussian distribution of CDR3 length profiles.

Owing to the complexity and diversity of the immune system, immunogenetics represents one of the greatest challenges for data interpretation: a large biological expertise, a considerable effort of standardization, and the elaboration of an efficient system for the management of the related knowledge were required. To answer that challenge, IMGT^®^, the international ImMunoGeneTics information system^®^(http://www.imgt.org), was created in 1989 by one of the authors (146). Overtime, it developed standards that, since 1995, have been endorsed by the World Health Organization-International Union of Immunological Societies (WHO-IUIS) Nomenclature Committee and by the WHO-International Nonproprietary Names (INN) (172–175). IMGT^®^ comprises seven databases (sequence, gene, and structure databases), 17 online tools and more than 15,000 pages of web resources. Among the databases, IMGT/LIGM-DB, the database for nucleotide sequences (170,685 sequences from 335 species as of July 2013) and IMGT/GENE-DB, the gene database (3,081 genes and 4,687 alleles) are of great interest for repertoire analysis. Freely available since 1997, IMGT/V-QUEST is an integrated system for the standardized analysis of collections of IG and TR rearranged nucleotide sequences (176, 177). A high-throughput version, IMGT/HighV-QUEST (148), has been released in 2010 for the analysis of long IG and TR sequences from NGS using the 454 Life Sciences technology. In the same line, other analysis tools are becoming available showing the renewed interest for repertoire analyses and modeling consecutive to NGS technology developments (178–181).

Altogether, these efforts highlight the relevance of developing more efficient and powerful technologies for the evaluation of repertoire diversity. Notably, two successful French biotech companies (TcLand, Nantes; ImmunID, Grenoble) were created in the field of repertoire analysis, using different technologies. In collaboration with ImmunID, we have proposed a novel strategy for statistical modeling of T lymphocyte repertoire data obtained in humans and humanized mice. With this model, we revealed that half of the human TRB repertoire, in terms of proportion of TRBV-TRBJ combinations, is genetically determined, the other half occurring stochastically (182). In addition, the biotechnology company “Adaptive” and the “Repertoire 10K (R10K) Project” have been recently founded by researchers respectively from the Fred Hutchinson Cancer Research Center (Seattle and Washington) and the HudsonAlpha Institute (Huntsville). Both have developed platforms (immunoSEQ^®^, iRepertoire^®^) providing researchers with a global analysis of the T or B cell receptor sequence repertoires (183). However, despite the power of this technology, studies are still limited by the ability to process the complexity of the information provided. Specific software developments for the automatic treatment and annotation of IG and TR sequences and the statistical modeling of repertoire diversity can still be improved.

#### Multivariate analysis

As mentioned above, the PANAMA-Blot technique also includes statistical analysis of the data. Multi-parametric analysis was introduced to compare the global reactivity of antibodies of different individuals in different groups with a given antigenic extract. This analysis has been successfully implemented to identify reactivity patterns specific for a given pathology or clinical status (10–12, 14, 15, 184). Similarly, multi-parametric analysis was also applied to TRBV spectratype analysis in an experimental cerebral malaria model (107).

Hierarchical clustering or classification algorithms have become very popular with the growing of micro-array-based transcriptome analysis. Although still uncommon for immune repertoire analysis, such approaches have been employed to categorize large sets of repertoire data without *a priori* (17, 102, 107).

#### Diversity indices

The concept of immune repertoire has been devised to describe the diversity of cells involved in the immune system of an individual (1). As described above, different scoring systems were developed to assess this diversity, some are heuristics but others have been borrowed from theoretical ecology and evolution. As reviewed by Magurran (185), the Shannon entropy, introduced by Claude Shannon in 1948 for the information theory, is the most used because it not only integrates the number of different species but also the relative proportion of each of these species. In 1961, Alfred Rényi generalized this entropy to a family of functions, like Species Richness, Simpson, Quadratic, and Berger–Parker indices, for quantifying the diversity, the uncertainty or randomness of a system. Most of these indices are implemented in the free software application Estimates (http://purl.oclc.org/estimates) (186). Altogether, these diversity indices constitute a collection of tools with their own sensitivity to the variety and the relative abundances of the species that are perfectly suitable for assessing immune repertoire diversity. Indeed, the very famous index of variability proposed by Kabat and Wu (187) corresponds to the ratio of Species Richness and Berger–Parker indices. In 1990, Jores et al. showed that the resolving power of this Wu-Kabat variability coefficient can be enhanced by increasing the weight on the frequency distribution of the amino acids in the formula (188). This approach inspired Stewart et al. (189) to use the Shannon entropy to demonstrate that TR amino acid composition is significantly more diverse than that of IG. In the same way, CDR3 spectratyping data can be analyzed using the relative abundance of each peak within CDR3 length global distribution. By doing so, we adjusted the original Shannon entropy, making it reaching its maximum for a Gaussian distribution, to compare the CDR3 length diversity of splenic IgM, IgD, and IgT in infected Teleost Fish (131). Recently, the Gini index, used in ecology or economics to measure the equality of distributions, was applied to individual TR clones and compared naive and memory repertoires (190). The development of deep sequencing techniques ignited a renewed interest in IG/TR repertoire. Indeed, several studies used high-throughput analysis to describe TR repertoire of key T cell subsets in human peripheral blood (115, 126, 191). This approach assessing the repertoire diversity from the relative abundance of each species in the global distribution can be decomposed hierarchically into components attributable, respectively, to variations in TRBV-TRBJ combinations and in CDR3-length (113, 117). However, most of these studies have been limited to the counting of the observed unique clonotypes. Beside the species richness, ecology-derived indices have also been applied to assess and compare immune repertoire diversity. Föhse et al. (119) used the Morisita-Horn similarity index to compare regulatory T cell repertoires between several lymphoid organs. In addition, Simpson diversity index, associated with Shannon entropy, was used to monitor TR repertoire diversity of HIV-specific CD8 T cells during antiretroviral therapy (192) but also to quantify TR repertoire recovery in the blood after allogeneic hematopoietic stem cell transplantation (128). In the same manner, Koning et al. (193) used Shannon’s and Simpson’s indices to show the role for the peptide component of the peptide-MH1 complex on the molecular frontline of CD8^+^ T cell–mediated immune surveillance, by comparing the repertoire diversity of CD8^+^ T cell populations directed against a variety of epitopes. In parallel, using Simpson’s index as a metric allowed Johnson et al. (194) to model mathematically the naïve CD4 T cell repertoire contraction with age leading them to conclude that diversity plummet observed around the age of 70 could be correlated to cell-intrinsic mutations affecting cell division rate or death.

### Modeling strategies

Modeling approaches have a strong tradition in immunology, usually at the boundary with other disciplines such as physics (195). Before deep sequencing data was available, general design principles were proposed as desirable features of immune repertoires, with implications for the observed repertoire diversity and dynamics (196–198). Many efforts have involved the modeling of immune cell dynamics and the effects of antigens on repertoire diversity, using differential equations descriptions of the population dynamics (199–201). Recognition in the immune system is often studied both theoretically and experimentally by probing the dynamics of cells with a specific type of receptor with respect to infections (202). Alternatively one can look at the response of a small set of chosen receptors to a specific pathogenic challenge, or careful biochemical investigation of particular receptor/antigen pairs (203, 204). Much work has been devoted to systems-biology approaches to signal processing in immune cells, as reviewed in Germain et al. (205) and Emonet and Altan-Bonnet (206). Here we focus on approaches inspired by recent advances in sequencing technologies (112, 113, 115, 116, 125, 191, 207, 208) that have opened the way for data-driven modeling of the immune repertoires and interactions between receptors and antigen.

A common modeling approach for describing receptors at the amino acid level is to choose a relevant interaction parameter (e.g., chemical affinity or hydrophobicity) and assign it a simplified digit-string representation (209). These methods are extensions of the string model, which describes both receptor and epitopes as strings of length L, with values chosen from natural numbers, and quantify their interaction by the match between the two strings (197, 210, 211). Such quantitative, physically inspired descriptions of immune receptors, despite the arbitrary choice of interaction coordinates, have proven a valuable first step in statistically describing recognition in T cells (195, 212–215). Recently, lower hydrophilicity of regulatory vs. conventional T cells was suggested from CDR3 sequencing (216).

High-throughput sequencing of immune receptors raises specific challenges compared to traditional genomic sequencing. It is harder to distinguish sequencing errors from new polymorphisms, since no corresponding pre-existing sequence exists. One of the most interesting regions when studying diversity is the CDR3 with its many insertions and deletions added to the germline sequence. These regions are often hard to align to the genomic templates, or with each other (217). Therefore, extra care is needed when generating and analyzing sequence data. Not all sequencing technologies are equally good for all purposes (218): while 454 sequencing gives longer reads than Illumina it is known to have a greater probability of frameshift errors. In addition, primer-dependent PCR amplification biases require that raw sequence counts be normalized using control experiments (112) in order to accurately report clone sizes, as demonstrated by spike-in experiments (219). In TR repertoire studies, this is circumvented by using 5′RACE which provides an unbiased amplification of fully rearranged sequences, as recently demonstrated for TRB V-(D)-J transcripts (191).

Despite sequencing issues, statistical algorithms are often able to extract information from the data. Many studies of diversity focus on the V, D, and J gene usage of each rearranged sequence. Algorithms and tools have been developed to rapidly identify the V, D, and J genes for massive numbers of sequences (148, 178, 181). In many cases however, the assignment of a D gene to each sequence read is unreliable if the D region is too short owing to extensive trimming. Mora et al. (217) learned from data and analyzed statistical models of the D gene flanked by its junctions. These models are based on the principle of maximum entropy and make minimal assumptions about the mechanisms of diversity – they only rely on the observed frequencies of amino acid pairs along the sequence. These models were used to describe global features of the sequence ensemble, such as the probability distribution following Zipf’s law (220) – the observation that the probability of sequences is inversely proportional to their frequency-rank, or the observation of peaks of frequency in sequence landscape as possible signatures of past pathogenic challenges. Recently, the estimation of repertoire diversity and clonal size distribution were analyzed by Poisson abundance models (221) and simple bivariate-Poisson-lognormal (BPLN) parametric model for fitting and analyzing TR repertoire data was proposed (222). Similarly, network analysis of IG repertoire from Weinstein et al. study revealed the possibility to identify subgroups of individuals on the basis of IG network similarity (223).

The task of characterizing the CDR3 at the nucleotide level is made difficult by the fact that a deterministic assignment of the V-(D)-J recombination process is impossible, because any given sequence can be generated by many possible recombination processes. A previous study proposed a probabilistic model of nucleotide trimming of rearranged TR genes derived from a benchmark data set of TRA and TRG V-(D)-J junctions obtained by comparison to the germline genes in the IMGT^®^ tools (224). Recently a statistical method based on the expectation-maximization algorithm was proposed to circumvent this issue and to extract the statistical properties of junctional diversity accurately from data (124). Applying it to human non-productive DNA sequences gave insight into a universal generation mechanism, reproducible from individual to individual. It was shown that each sequence could potentially be generated by the equivalent of ∼30 equally likely ways by convergent recombination. This method showed that the potential diversity of the recombination machinery was equivalent to ∼10^14^ *equally likely* sequences (and a practically infinite total number of possible sequences), much more than the estimated 10^12^ T cells that a single human body can hold. The frequencies of the V, D, and J genes is non-uniform, even at the level of recombination, suggesting underlying physical mechanisms at work. Ndifon et al. (125) proposed a polymer model that accounts for the likelihood of connecting given genomic fragments, giving insight into the mechanistic process.

One of the ultimate goals of deep repertoire sequencing is to find signatures of the repertoire’s response to its antigenic environment. A combination of clustering methods and tree reconstruction techniques have been developed (225, 226) to identify lineages in B cells and study the response to pathogenic challenges. Statistical methods have been devised to detect and quantify the extent of antigen-driven selection acting on B cells, by analyzing the patterns of hypermutations in a Bayesian framework, with applications to deep sequencing data (227, 228).

A lot remains to be done in terms of both data-driven and small-scale models of repertoire-antigen interactions. Ultimately, a close collaboration and development of experimental techniques and models can shed light on how selection at different stages shapes the repertoire, how affinity maturation changes the diversity and the link between sequence diversity and function.

### Future prospects of biomathematical analysis of repertoire data

One of the current challenging issues in antigen-specific repertoire analysis is the development of relevant statistical analysis strategies. Biologists are usually keen on parametric tests, such as ANOVA, *t*-test, Fischer’s test, among others. However, such statistical methods assume that the inherent probability distribution of the observed variable follows a normal distribution. Rock et al. (229) described that the distribution of the TR diversity is far from following this distribution, thus they proposed the use of non-parametric tests. Nevertheless, different groups are dealing with this issue in order to determine the relevant way to analyze repertoire diversity data and to propose new biostatistics strategies, including principal component analysis, discriminant analysis, hierarchical clustering, specific statistics (164, 169).

In fact, the traditional use of statistics in biology aims at the falsification of a defined hypothesis, i.e., at validating significant differences between defined situations. The recent development of “systems immunology” reverses this point of view and establishes a new usage of multi-parametric statistical approaches to represent the biological data by projections and “landscapes” in the N-dimensional space of considered parameters (230). Thus, the traditional description of separate repertoires for distinct cell subsets defined from a few markers is being replaced by overlapping clouds of data, setting the limits of the different classification groups (tissue of origin, infection contexts, combination of marker expression, repertoire expression…). Moreover, repertoire diversity technologies can now be combined to complementary approaches to decipher the complexity of lymphocyte populations, such as microwell array cell culture and high-resolution imaging (231), mass cytometry (232, 233), cellular barcoding (234), intravital imaging (235, 236), single-cell gene expression (237). In addition, high-throughput repertoire descriptions will enrich mathematical and computer models of lymphocyte repertoire diversity and dynamics such as those proposed by Mehr (238), Ciupe et al. (239), or Stirk et al. (240).

As advocated by others, the concepts developed by systems biology, such as the signatures emerging from clustering and the modularity regulating gene networks, will probably need to be adapted to the constraints of immunology data (241). However, this is probably through this kind of representation that global analysis of immune repertoires will have to be addressed (242).

The upcoming challenge is now to merge data produced through the different technological approaches available to achieve full integration of these data and make them available for interactive meta-analysis. This necessitates more than the simple juxtaposition of annotated raw data but rather requires (1) the codification and standardization of this multi-level data and (2) the integration of complexity science into immunology. Along this line, recent developments of multi-parametric flow cytometry naturally led to systematic clustering and multivariate statistical analysis approaches for searching functional signatures (2, 232, 233, 243–245).

## Conflict of Interest Statement

The authors declare that the research was conducted in the absence of any commercial or financial relationships that could be construed as a potential conflict of interest.
